# Advisory Committee on Immunization Practices Recommended Immunization Schedule for Adults Aged 19 Years or Older — United States, 2023

**DOI:** 10.15585/mmwr.mm7206a2

**Published:** 2023-02-10

**Authors:** Neil Murthy, A. Patricia Wodi, Veronica McNally, Sybil Cineas, Kevin Ault

**Affiliations:** ^1^Immunization Services Division, National Center for Immunization and Respiratory Diseases, CDC; ^2^Fanny Strong Foundation, West Bloomfield, Michigan; ^3^The Warren Alpert Medical School of Brown University, Providence, Rhode Island; ^4^University of Kansas Medical Center, Kansas City, Kansas.

At its October 2022 meeting, the Advisory Committee on Immunization Practices* (ACIP) approved the Recommended Adult Immunization Schedule for Ages 19 Years or Older, United States, 2023. The 2023 adult immunization schedule summarizes ACIP recommendations, including several changes to the cover page, tables, notes, and appendix from the 2022 immunization schedule.^†^ This schedule can be found on the CDC immunization schedule website (https://www.cdc.gov/vaccines/schedules). Health care providers are advised to use the cover page, tables, notes, and appendix together to determine recommended vaccinations for patient populations. This adult immunization schedule is recommended by ACIP (https://www.cdc.gov/vaccines/acip) and approved by CDC (https://www.cdc.gov), the American College of Physicians (https://www.acponline.org), the American Academy of Family Physicians (https://www.aafp.org), the American College of Obstetricians and Gynecologists (https://www.acog.org), the American College of Nurse-Midwives (https://www.midwife.org), the American Academy of Physician Associates (https://www.aapa.org), the American Pharmacists Association (https://www.pharmacist.com), and the Society for Healthcare Epidemiology of America (https://shea-online.org).

ACIP’s recommendations for the use of each vaccine are developed after in-depth reviews of vaccine-related data, including disease epidemiology and societal impacts, vaccine efficacy and effectiveness, vaccine safety, quality of evidence, feasibility of program implementation, and economic analyses of immunization policy (*1*). The adult immunization schedule is published annually to consolidate and summarize updates to ACIP recommendations on vaccination of adults and to assist health care providers in implementing current ACIP recommendations. The use of vaccine trade names in this report and in the adult immunization schedule is for identification purposes only and does not imply endorsement by ACIP or CDC.

For further guidance on the use of each vaccine, including any changes that might occur after annual publication of the adult immunization schedule, health care providers are referred to the respective ACIP vaccine recommendations at https://www.cdc.gov/vaccines/hcp/acip-recs. If errors or omissions are discovered within the schedule, CDC will post revised versions on the CDC immunization schedule website.^§^ Printable versions of the 2023 adult immunization schedule and instructions for ordering hard copies of the schedule are available on the immunization schedule website (https://www.cdc.gov/vaccines/schedules).

## Changes in the 2023 Adult Immunization Schedule

Vaccine-specific changes in the 2023 immunization schedule for adults aged ≥19 years include new or updated ACIP recommendations for influenza vaccines (*2*) and pneumococcal vaccines (*3*). Additional information was added for the measles, mumps, and rubella vaccine (MMR), meningococcal vaccine, and recombinant zoster vaccine (RZV) sections. The hepatitis B vaccine (HepB) section was rearranged and revised to improve clarity in the language, and minor edits were made to the tetanus, diphtheria, and acellular pertussis vaccination (Tdap) notes to improve readability. In addition, COVID-19 vaccines have been added to the Tables and to the Notes sections summarizing ACIP recommendations. A new poliovirus vaccination section was also added to the Notes section to describe the use of inactivated poliovirus vaccine (IPV) in adults who are at increased risk for exposure to polioviruses. Changes were also made to the appendix section to improve clarity in the language.

### Cover page

• The American Pharmacists Association has been added as a partner organization approving the adult immunization schedule.

• A newly recommended HepB vaccine (PreHevbrio) and a newly recommended MMR vaccine (Priorix) have been added to the table of vaccine abbreviations and trade names.

• COVID-19 vaccines have been added to the table of vaccine abbreviations and trade names. ACIP has developed new abbreviations for the COVID-19 vaccine products. These abbreviations contain information on the vaccine’s valency (i.e., monovalent versus bivalent, indicated by “1v” and “2v,” respectively) and vaccine platform (mRNA versus acellular protein subunit, or “aPS”).

• The pneumococcal conjugate vaccines PCV15 and PCV20 have been combined into one row in the table of vaccine abbreviations and trade names.

• The language in the injury claims section has been modified to indicate which vaccines are covered by the National Vaccine Injury Compensation Program and which vaccines are covered by the Countermeasures Injury Compensation Program.

### Table 1 (Routine Immunization Schedule)

• **COVID-19 row:** The COVID-19 vaccine row is a new addition to the tables this year. The color of this row is yellow, indicating that COVID-19 vaccination is now routinely recommended for all adults. The text overlay states, “2- or 3-dose primary series and booster (See Notes).”

• **MMR row**: Overlaying text has been added to the column for persons aged ≥65 years referring providers to the notes for vaccination considerations for health care personnel in this age group.

• **Hepatitis A row**: The overlaying text has been updated to “2, 3, or 4 doses depending on vaccine,” to account for the possibility of an accelerated Twinrix series requiring 4 doses.

### Table 2 (Immunization by Medical Indication Schedule)

• **COVID-19 row**: The COVID-19 vaccine row is a new addition to the tables this year. The color of this row is yellow, indicating that COVID-19 vaccination is now routinely recommended for adults with any of the medical conditions or other indications listed. The text overlay for the immunocompromised and HIV infection columns states, “See Notes,” referring providers to the notes for specific recommendations for this population.

• **Hepatitis A row**: The overlaying text has been updated to “2, 3, or 4 doses depending on vaccine,” to account for the possibility of an accelerated Twinrix series requiring 4 doses.

### Vaccine Notes

The notes for each vaccine are presented in alphabetical order. Edits have been made throughout the Notes section to harmonize language between the child and adolescent and the adult immunization schedules to the greatest extent possible.

• **COVID-19**: A new section was added to provide additional details for use of COVID-19 vaccines. The “Routine vaccination” section describes the primary series recommendations for the general population. The “Special situations” section describes the primary series recommendations for persons who are moderately or severely immunocompromised. Hyperlinks have been provided referring health care providers to the latest guidance for booster dose recommendations in both populations, and to the recommendation for persons who received the Janssen (Johnson & Johnson) COVID-19 vaccine. Additionally, hyperlinks to the current COVID-19 vaccination schedules, use of COVID-19 preexposure prophylaxis in persons who are moderately or severely immunocompromised, as well as Emergency Use Authorization indications for COVID-19 vaccines, have been added.

• **HepB**: In the “Routine vaccination” section, PreHevbrio was added to the description of the 3-dose series, and information on the 4-dose series for persons on hemodialysis was moved to the “Special situations” section. HepB vaccination continues to be universally recommended for all adults aged 19–59 years. Language has been added stating that persons aged ≥60 years with known risk factors for hepatitis B virus infection should complete a HepB vaccination series, whereas persons aged ≥60 years without known risk factors for hepatitis B virus infection may complete a HepB vaccination series.

• **Influenza**: Information was added to the routine vaccination section for persons aged ≥65 years stating that any one of quadrivalent high-dose inactivated influenza vaccine, quadrivalent recombinant influenza vaccine, or quadrivalent adjuvanted inactivated influenza vaccine is preferred for this age group. A hyperlink to the 2022–23 influenza recommendations and a bullet for the 2023–24 influenza recommendations were added. In the “Special situations” section, guidance for close contacts of severely immunocompromised patients who require a protected environment was added. In addition, the text describing guidance for persons with egg allergy who have experienced any symptom other than hives was moved from the appendix to the “Special situations” section.

• **MMR**: In the “Special situations” section, a hyperlink was provided that describes the recommendation for additional doses of MMR vaccine (including the third dose of MMR vaccine) in the context of a mumps outbreak setting.

• **Meningococcal**: In the “Special situations” section for meningococcal serogroup B vaccine, guidance was added stating that if the third dose of Trumenba is administered earlier than 4 months after the second dose, a fourth dose should be administered ≥4 months after the third dose.

• **Pneumococcal**: The section has been substantially updated to reflect ACIP’s new recommendations for the use of PCV15 and PCV20 in persons who previously received pneumococcal vaccines. In addition, a hyperlink to the CDC app that can be used to determine a patient’s pneumococcal vaccination needs has been included.

• **Poliovirus**: A new section was added summarizing poliovirus vaccination recommendations for adults. Although routine vaccination of adults residing in the United States is not necessary, the “Special situations” section describes the use of IPV in adults who are at increased risk for exposure to poliovirus.

• **Tdap**: Minor changes were made to the “Special situations” section to improve clarity in the language.

• **Zoster**: The “Routine vaccination” section was revised to clarify that serologic evidence of prior varicella is not necessary for zoster vaccination and to provide guidance for situations in which serologic evidence of varicella susceptibility becomes available. The “Special situations” section was updated to provide guidance for persons with immunocompromising conditions who do not have a documented history of varicella, varicella vaccination, or herpes zoster. In addition, minor changes were made to the immunocompromising conditions bullet to clarify that this includes persons with HIV regardless of CD4 count.

### Appendix (Contraindications and Precautions)

• The header of the “Contraindications” column was changed to “Contraindicated or not recommended.”

• **Influenza (egg-based) row**: The information for persons with history of egg allergy was moved from the precautions column to the influenza vaccination notes section.

• **HepB row**: The language regarding the use of Heplisav-B and PreHevbrio in pregnant persons was modified. The language now states that “Heplisav-B and PreHevbrio are not recommended because of lack of safety data in pregnant persons. Use other hepatitis B vaccines if HepB is indicated.” A footnote providing information on the pregnancy exposure registries for persons who were inadvertently vaccinated with Heplisav-B and PreHevbrio while pregnant was added.

• **Human papillomavirus row**: The language regarding the use of human papillomavirus (HPV) vaccination among pregnant persons was modified. The language now states, “pregnancy: HPV vaccination not recommended.”

## Additional Information

The Recommended Adult Immunization Schedule, United States, 2023, is available at https://www.cdc.gov/vaccines/schedules/hcp/adult.html and in the *Annals of Internal Medicine* (https://www.acpjournals.org/doi/10.7326/M23-0041). The full ACIP recommendations for each vaccine are also available at https://www.cdc.gov/vaccines/hcp/acip-recs/index.html. All vaccines identified in Tables 1 and 2 (except PCV20 and RZV) also appear in the Recommended Immunization Schedule for Children and Adolescents, United States, 2023 (https://www.cdc.gov/vaccines/schedules/hcp/imz/child-adolescent.html). The notes and appendices for vaccines that appear in both the adult immunization schedule and the child and adolescent immunization schedule have been harmonized to the greatest extent possible.

## Footnotes

* Recommendations for routine use of vaccines in adults are developed by ACIP, a federal advisory committee chartered to provide expert external advice and guidance to the CDC director on use of vaccines and related agents for the control of vaccine-preventable diseases in the civilian population of the United States. Recommendations for routine use of vaccines in adults are harmonized to the greatest extent possible with recommendations made by the American Academy of Pediatrics, the American Academy of Family Physicians, and the American College of Obstetricians and Gynecologists. ACIP recommendations approved by the CDC director become agency guidelines on the date published in the *Morbidity and Mortality Weekly Report*. Additional information about ACIP is available at https://www.cdc.gov/vaccines/acip/.

† Past immunization schedules are available at https://www.cdc.gov/vaccines/schedules/hcp/schedule-related-resources.html#accordion-2-collapse-3.

§ CDC encourages organizations to use syndication as a more reliable method for displaying the most current and accurate immunization schedules on an organization’s website rather than copying these schedules to their websites. Use of content syndication requires a one-time step that ensures an organization’s website displays current schedules as soon as they are published or revised; instructions for the syndication code are available on CDC’s website (https://www.cdc.gov/vaccines/schedules/resource-library/syndicate.html). CDC also offers technical assistance for implementing this form of content syndication (requests can be e-mailed to ncirdwebteam@cdc.gov).
